# Intestinal microsporidiosis among HIV/AIDS patients receiving antiretroviral therapy in Sana’a city, Yemen: first report on prevalence and predictors

**DOI:** 10.1186/s12879-021-07009-3

**Published:** 2022-01-04

**Authors:** Kwkab A. R. Al-Brhami, Rashad Abdul‑Ghani, Salah A. Al-Qobati

**Affiliations:** 1 Department of Medical Parasitology, Faculty of Medicine and Health Sciences, Sana’a University, Sana’a, Yemen; 2 Tropical Disease Research Center, Faculty of Medicine and Health Sciences, University, of Science and Technology, Sana’a, Yemen

**Keywords:** Intestinal microsporidiosis, HIV/AIDS, Immunodeficiency, Prevalence, Predictors, Yemen

## Abstract

**Background:**

Intestinal microsporidiosis is an opportunistic infection associated with persistent diarrhea among HIV/AIDS patients. In Yemen, however, its epidemiology is unknown. Therefore, this study determined its prevalence and predictors among HIV/AIDS patients receiving antiretroviral therapy (ART) in Sana’a city, the capital of Yemen.

**Methods:**

This cross-sectional study included 402 patients receiving ART at Al-Jomhori Educational Hospital in Sana’a from November 2019 to December 2020. Data about demographics, clinical characteristics and risk factors were collected using a pre-designed questionnaire. Stool samples were collected and examined for microsporidian spores using the Gram-chromotrope Kinyoun staining. Blood samples were also collected and used for CD4 cell counting by flow cytometry. Univariate analysis was used to test the association of patients’ characteristics and risk factors with intestinal microsporidiosis. Multivariable logistic regression was then used to identify the independent predictors of infection. Statistical significance was considered at *P*-values < 0.05.

**Results:**

Intestinal microsporidiosis was prevalent among 14.2% (57/402) of HIV/AIDS patients and was significantly associated with diarrhea (OR 3.4, 95% CI 1.7–6.6; *P* = 0.001). The significant independent predictors of infection were < 200 CD4 cells/µl (AOR 3.2, 95% CI 1.5–6.9; *P* = 0.003), not washing hands after contacting soil (AOR 2.5, 95% CI 1.1–5.4; *P* = 0.026) and before eating (AOR 3.1, 95% CI 1.5–6.4; *P* = 0.003), eating unwashed raw produce (AOR 2.5, 95% CI 1.2–5.3; *P* = 0.017) and absence of indoor latrines (AOR 6.2, 95% CI 1.5–25.9; *P* = 0.012).

**Conclusions:**

The prevalence of intestinal microsporidiosis among HIV/AIDS patients in Sana'a is high and comparable to that reported from several other countries, being prevalent among approximately 14.0% of patients and significantly associated with diarrhea. It could be predicted among patients who have < 200 CD4 cells/µl, have poor hand hygiene after contacting soil and before eating, usually eat unwashed raw produce, or do not possess indoor latrines. Large-scale studies on its epidemiology and predictors among HIV/AIDS patients across the country are warranted.

## Introduction

Microsporidia are a group of unicellular, spore-forming parasites closely related to fungi that infect a wide spectrum of invertebrate and vertebrate hosts [1, 2]. Human microsporidiosis was first reported in the late 1920s as reviewed by Weiss et al. [3], but human infections have been rarely reported before the discovery of the human immunodeficiency virus (HIV)/acquired immunodeficiency syndrome (AIDS) [4]. Since the AIDS pandemic in the early 1980s, microsporidia have emerged as important opportunistic parasites affecting immunocompetent individuals and immunocompromized patients, particularly AIDS patients, in developed and developing countries [3, 5]. The most frequent causative agents of intestinal microsporidiosis in immunocompromized patients are *Enterocytozoon bieneusi* and *Encephalitozoon intestinalis* [6]. Humans acquire infection with microsporidia through several routes, including the ingestion or inhalation of spores besides direct and indirect zoonotic transmission [5, 7]. Vertical transmission has been reported in experimental animals, but no congenitally transmitted cases have been confirmed in humans [8]. Although intestinal microsporidiosis is usually asymptomatic or self-limiting in immunocompetent individuals, it can be severe and life-threatening in immunocompromized patients, particularly AIDS patients having a cluster of differentiation 4 (CD4+) T cells (also known as CD4 cells) of < 100 cells/μl of blood [9]. Such patients are prone to develop chronic and debilitating diarrhea and other gastrointestinal manifestations, fever, weight loss and malabsorption syndrome [10].

HIV is a retrovirus that destroys activated CD4 cells, which play a major role in the regulation of humoral and cell-mediated immune responses, leading to infection with opportunistic pathogens that usually appear when the CD4 cell count declines below 200 cells per µl of blood as in AIDS patients [11]. By 2016, 36.7 million people have been estimated to be living with HIV worldwide, 1.8 million people became newly infected with the virus and 1.0 million people lost their lives because of AIDS [12]. In Yemen, the first HIV case was detected in 1987, and the National AIDS Control Program (NACP) was then established within the organogram of the Ministry of Public Health and Population to monitor and control HIV/AIDS in the country by providing counseling, testing and antiretroviral therapy (ART) services [13]. According to the NACP, a low HIV prevalence of 0.2% was estimated in 2011 in the country [14]. In 2016, 9900 people living with HIV have been estimated in the general population in the country despite reporting only 2701 cases [15].

Different laboratory techniques have been used to identify microsporidian spores in various clinical specimens, including light and fluorescence microscopy, transmission electron microscopy (TEM) as well as immunological and molecular techniques [16]. TEM is the gold standard technique to identify the specific species; however, it is time-consuming, expensive and cannot be performed on a large number of samples [17]. Polymerase chain reaction (PCR)-based techniques have improved the sensitivity and specificity of detection to the species level [16]. However, such techniques are not used routinely because they are time-consuming, labor-intensive and costly in addition to the difficulty in isolating the nucleic acids from the spores and the presence of PCR inhibitors in stool samples [6, 18]. Therefore, intestinal microsporidiosis is commonly diagnosed by the microscopical detection of spores in stool smears stained with Weber’s modified trichrome (WMT) technique [3, 19–21]. Several modifications have been developed, with Gram-chromotrope staining being the much-improved differential technique for identifying the microsporidian spores [22, 23]. Gram-chromotrope Kinyoun (GCK) staining technique was later developed as a modification of the Gram-chromotrope technique with high specificity and sensitivity for identifying microsporidian spores [24].

On a global scale, the prevalence of intestinal microsporidiosis in HIV-infected patients varies considerably across countries and ranges from below 1.0% to over 80.0%, predominantly in Southeast Asia, Middle East, Europe, Africa and Latin America [25]. It has been reported among 10–50% of HIV-infected patients with chronic diarrhea [25, 26]. In non-HIV-infected individuals, most infections occur in children, the elderly, travelers and organ transplant recipients [3]. In Yemen, one of the least-developed countries, the epidemiology of intestinal microsporidiosis is unknown. With the lack of published studies on its epidemiology among HIV/AIDS patients in the country, this study aimed to determine the prevalence of intestinal microsporidiosis, to identify its predictors and to assess its association with certain clinical manifestations among HIV/AIDS patients in Sana’a city.

## Methods

### Study design and area

This hospital-based, cross-sectional study was conducted in the ART clinic of the NACP at Al-Jomhori Educational Hospital in Sana'a city in the period from November 2019 to December 2020. Sana'a is located at the coordinates of 15° 20′ 54″ N and 44° 12′ 23 E in north of Yemen. It has an estimated population of over 2.5 million people [27]. It is noteworthy that only one ART clinic offers treatment services to HIV/AIDS patients in the city.

### Study population

HIV/AIDS patients receiving ART in the clinic of the NACP in Sana’a were recruited for this study irrespective of their gender or age. Patients were included if confirmed positive for HIV by enzyme-linked immunosorbent assay (ELISA) or PCR. Patients with other immunocompromizing conditions, such as cancer patients undergoing chemotherapy, organ transplant recipients, or hemodialysis patients, and those who refused to give written informed consent to participate were excluded.

### Sample size and sampling strategy

The minimum sample size required was calculated to be 384 using EpiInfo software, version 2.3.1 (Centers for Disease Control and Prevention, Atlanta, USA). Because of the lack of previous studies on intestinal microsporidiosis among this population category, the calculation was based on the following parameters: an expected prevalence of 50.0%, a confidence level of 95.0% and a precision of 5.0%. However, 402 patients were included in this study by simple random sampling from the HIV/AIDS patients registered in the ART clinic of the NACP.

### Data collection

Data about demographics, clinical characteristics and potential risk factors associated with intestinal microsporidiosis were collected using a pre-designed, structured questionnaire through face-to-face interviews. Patients were considered diarrheic if they had reported: *the passage of three or more loose or liquid stools per day or more frequent passage than is normal for the individual* according to WHO criteria [28], either on admission or one month before sample collection.

### Sample collection

Fresh stool samples were collected into clean, dry, leak-proof and pre-labeled containers and were preserved in 10% formalin until examined. In addition, about 3 ml of blood samples were collected by aseptic venipuncture into pre-labeled tubes with ethylenediaminetetraacetic acid (EDTA) for CD4 cell counting.

### Detection of microsporidian spores

Stool specimens were processed and examined at the Parasitology Laboratory at the Faculty of Medicine and Health Sciences, Sana’a University. Thin smears were prepared from unconcentrated stool [29] on pre-labeled glass slides, which were then thoroughly dried, fixed with absolute methanol and left to air-dry. The dried smears were stained with the GCK technique [24]. The stained smears were examined for microsporidian spores under the oil-immersion objective lens of a light microscope. The criteria used to identify the microsporidian spores were the detection of pinkish-blue ovoid structures with blue walls and encircled with deeply blue belt-like structures against a pale pink background [24]. At least 100 oil-immersion fields were examined before recoding the result as being negative for microsporidian spores.

### CD4 cell counting

Twenty-five microliters of EDTA-anticoagulated blood were incubated with specific monoclonal antibodies labeled with a fluorescent dye. Blood was then used to count CD4 cells by flow cytometry using Pima™ analyzer (Alere Technologies GmbH, Jena, Germany) as per the instructions of the manufacturer.

### Data analysis

Data were analyzed using the IBM SPSS Statistics for Windows, Version 22.0 (IBM Corp., Armonk, NY, USA). Categorical variables were expressed as frequencies and proportions, while continuous variables were expressed as median ± interquartile range (IQR) for non-normally distributed data. The prevalence of intestinal microsporidiosis was reported with its corresponding 95.0% confidence interval (CI). Pearson's chi-square or Fisher’s exact test, whichever suitable, was used to test the association of the independent variables (demographics, clinical characteristics and risk factors) with intestinal microsporidiosis as the dependent outcome in a univariate analysis. The odds ratios (ORs) and 95.0% CIs for the association of the independent variables with intestinal microsporidiosis were reported. A multivariable logistic regression analysis of the significant predictors in the univariate analysis was then used to identify the independent predictors of intestinal microsporidiosis together with their adjusted ORs (AORs) and 95.0% CIs. *P*-values < 0.05 were considered statistically significant.

## Results

### Demographics of HIV/AIDS patients

Table 1 shows that the majority of HIV/AIDS patients in Sana'a city were males (77.9%), aged less than 40 years (64.9%) with a median age of 37.0 ± 15.0 years (range: 5–80), urban residents (55.8%) and living within households of 5 members or more (60.4%) with a median household size of 7 ± 5 members. Approximately one-third of patients had primary education followed by those illiterate and who had secondary education (27.3% and 20.7%, respectively). More than half of patients were unemployed (56.2%). Day workers represented 18.7% of patients, while public service employees represented the least frequent category of patients (6.3%).

**Table 1:** Demographics of HIV/AIDS patients receiving ART in the clinic of NACP at Al-Jomhori Educational Hospital in Sana’a, Yemen (2019–2020)

Characteristics	*n*	(%)
Gender		
Male	313	(77.9)
Female	89	(22.1)
Age (years)		
< 40	261	(64.9)
≥ 40	141	(35.1)
Median ± IQR: 37.0 ± 15.0		
Range 5–80		
Residence^a^		
Rural	176	(44.2)
Urban	222	(55.8)
Household size (members)		
< 5	159	(39.6)
≤ 5	243	(60.4)
Median ± IQR: 7 ± 5		
Educational level^b^		
Illiterate	99	(27.3)
Informal education	10	(2.8)
Primary education	134	(36.9)
Secondary education	75	(20.7)
University and above	45	(12.4)
Occupation^c^		
Unemployed	222	(56.2)
Public service employee	25	(6.3)
Private sector employee	40	(10.1)
Day worker	74	(18.7)
Self-employed	34	(8.6)

The total number of patients included in the study was 402; ^a^four missing cases; ^b^39 missing cases; ^c^7 missing cases

*HIV* human immunodeficiency virus, *AIDS* acquired immunodeficiency syndrome, *ART* antiretroviral therapy, *NACP* National AIDS Control Program, *IQR* interquartile range

### Clinical characteristics of HIV/AIDS patients

Table 2 shows that 22.7% of HIV/AIDS patients had CD4 cell counts of < 200 cells/µl, with a median count of 355 ± 288 cells/µl. The clinical manifestations reported by patients were abdominal pain (32.8%), diarrhea (12.9%), nausea (22.6%) and vomiting (9.5%).

**Table 2:** Clinical characteristics of HIV/AIDS patients receiving ART in the clinic of NACP at Al-Jomhori Educational Hospital in Sana’a, Yemen (2019–2020)

Characteristics	*n*	(%)
CD4 cell count (/µl)^a^		
< 200	90	(22.7)
≥ 200	306	(77.3)
Median ± IQR: 355 ± 288		
Diarrhea		
Yes	52	(12.9)
No	350	(87.1)
Nausea		
Yes	91	(22.6)
No	311	(77.4)
Vomiting		
Yes	38	(9.5)
No	364	(90.5)
Abdominal pain		
Yes	132	(32.8)
No	270	(67.2)

The total number of patients included in the study was 402; ^a^six missing cases

*HIV* human immunodeficiency virus, *AIDS* acquired immunodeficiency syndrome, *ART* antiretroviral therapy, *NACP* National AIDS Control Program, *CD4* cluster of differentiation 4, *IQR* interquartile range

### Prevalence of intestinal microsporidiosis among HIV/AIDS patients

Out of 402 HIV/AIDS patients, 57 (14.2%; 95% CI 11.0–18.1) were positive for intestinal microsporidiosis.

### Association of intestinal microsporidiosis with demographics and clinical characteristics of HIV/AIDS patients

There was no statistically significant association between intestinal microsporidiosis and gender, age, residence, household size, literacy status, or employment status of HIV/AIDS patients in Sana’a city. On the other hand, intestinal microsporidiosis was significantly higher among HIV/AIDS patients having diarrhea (OR 3.4, 95% CI 1.7–6.6; *P* = 0.001) but was not significantly associated with other gastrointestinal conditions (Table 3).

**Table 3:** Association of intestinal microsporidiosis with demographics and clinical characteristics of HIV/AIDS patients receiving ART in the clinic of NACP at Al-Jomhori Educational Hospital in Sana’a, Yemen (2019–2020)

Variable	*N*	Intestinal microsporidiosis		*P*-value
		*n* (%)	OR (95% CI)	
Gender				
Male	313	41 (13.1)	Reference	0.301
Female	89	16 (18.0)	1.5 (0.8–2.7)	
Age (years)				
< 40	261	39 (14.9)	0.8 (0.5–1.5)	0.653
≥ 40	141	18 (12.8)	Reference	
Residence				
Rural	176	30 (17.0)	1.5 (0.8–2.6)	0.195
Urban	222	27 (12.2)	Reference	
Household size (members)				
< 5	159	16 (10.1)	Reference	0.059
≥ 5	243	41 (16.9)	1.8 (1.0–3.4)	
Literacy status				
Illiterate	99	17 (17.2)	1.3 (0.7–2.4)	0.508
Literate	264	37 (14.0)	Reference	
Employment status				
Unemployed	222	36 (16.2)	1.5 (0.8–2.7)	0.195
Employed	173	20 (11.6)	Reference	
Diarrhea				
Yes	52	16 (30.8)	3.4 (1.7–6.6)	0.001
No	350	41 (11.7)	Reference	
Nausea				
Yes	91	14 (15.4)	1.1 (0.6–2.2)	0.733
No	311	43 (13.8)	Reference	
Vomiting				
Yes	38	9 (23.7)	2.0 (0.9–4.6)	0.088
No	364	48 (13.2)	Reference	
Abdominal pain				
Yes	132	23 (17.4)	1.5 (0.8–2.6)	0.223
No	270	34 (12.6)	Reference	

*N* number examined, *n* number infected with intestinal microsporidiosis, *HIV* human immunodeficiency virus, *AIDS* acquired immunodeficiency syndrome, *ART* antiretroviral therapy, *NACP* National AIDS Control Program, *OR* odds ratio, *CI* confidence interval

### Predictors of intestinal microsporidiosis among HIV/AIDS patients

Having < 200 CD4 cells/µl (OR 2.7, 95% CI 1.5–5.0; *P* = 0.001), not washing hands after contact with soil (OR 3.1, 95% CI 1.5–6.4; *P* = 0.002), not washing hands before eating (OR 3.7, 95% CI 2.0–6.6; *P* < 0.001), eating unwashed raw produce (OR 2.1, 95% CI 1.2–3.7; *P* = 0.015) and absence of indoor latrines (OR 13.5, 95% CI 4.8–38.3; *P* < 0.001) were significantly associated with intestinal microsporidiosis among HIV/AIDS patients. In contrast, there was no statistically significant association between intestinal microsporidiosis and the source of drinking water, bathing and/or swimming outdoors, presence of animals in the house, or indiscriminate defecation (Table 4).

**Table 4:** Predictors of intestinal microsporidiosis among HIV/AIDS patients receiving ART in the clinic of NACP at Al-Jomhori Educational Hospital in Sana’a, Yemen (2019–2020)

Variable	*N*	Intestinal microsporidiosis		*P*-value
		*n* (%)	OR (95% CI)	
CD4 cell count (/µl)				
< 200	90	23 (25.6)	2.7 (1.5–5.0)	0.001
≥ 200	306	34 (11.1)	Reference	
Source of drinking water				
Bottled	150	17 (11.3)	Reference	0.238
Unbottled	252	40 (15.9)	1.5 (0.8–2.7)	
Bathing and/or swimming outdoors				
Yes	93	12 (12.9)	0.9 (0.4–1.7)	0.738
No	309	45 (14.6)	Reference	
Contact with soil				
Yes	287	46 (16.0)	2.4 (0.9–6.2)	0.083
No	67	5 (7.5)	Reference	
Washing hands after contact with soil				
Yes	133	11 (8.3)	Reference	0.002
No	155	34 (21.9)	3.1 (1.5–6.4)	
Washing hands before eating				
Yes	307	30 (9.8)	Reference	< 0.001
No	95	27 (28.4)	3.7 (2.0–6.6)	
Eating unwashed raw produce				
Yes	199	37 (18.6)	2.1 (1.2–3.7)	0.015
No	201	20 (10.0)	Reference	
Presence of domestic animals in the house				
Yes	178	22 (12.4)	0.8 (0.4–1.4)	0.390
No	224	35 (15.6)	Reference	
Presence of indoor latrines				
Yes	385	46 (11.9)	Reference	< 0.001
No	17	11 (64.7)	13.5 (4.8–38.3)	
Indiscriminate defecation				
Yes	162	24 (14.8)	1.1 (0.6–2.0)	0.877
No	182	25 (13.7)	Reference	

*N* number examined, *n* number infected with intestinal microsporidiosis, *HIV* human immunodeficiency virus, *AIDS* acquired immunodeficiency syndrome, *ART* antiretroviral therapy, *NACP* National AIDS Control Program, *CD4* cluster of differentiation 4, *OR* odds ratio, *CI* confidence interval

### Independent predictors of intestinal microsporidiosis among HIV/AIDS patients

Multivariable logistic regression analysis showed that having < 200 CD4 cells/µl (AOR 3.2, 95% CI 1.5–6.9; *P* = 0.003), not washing hands after contact with soil (AOR 2.5, 95% CI 1.1–5.4; *P* = 0.026), not washing hands before eating (AOR 3.1, 95% CI 1.5–6.4; *P* = 0.003), eating unwashed raw produce (AOR 2.5, 95% CI 1.2–5.3; *P* = 0.017) and absence of indoor latrines (AOR 6.2, 95% CI 1.5–25.9; *P* = 0.012) were independent predictors significantly associated with intestinal microsporidiosis among HIV/AIDS patients (Table 5).

**Table 5:** Independent predictors associated with intestinal microsporidiosis among HIV/AIDS patients receiving ART in the clinic of NACP at Al-Jomhori Educational Hospital in Sana’a, Yemen (2019–2020)

Variable	AOR	95% CI	*P*-value
Having < 200 CD4 cells/µl	3.2	1.5–6.9	0.003
Not washing hands after contact with soil	2.5	1.1–5.4	0.026
Not washing hands before eating	3.1	1.5–6.4	0.003
Eating unwashed raw produce	2.5	1.2–5.3	0.017
Absence of indoor latrines	6.2	1.5–25.9	0.012

*HIV* human immunodeficiency virus, *AIDS* acquired immunodeficiency syndrome, *ART* antiretroviral therapy, *NACP* National AIDS Control Program, *CD4* cluster of differentiation 4, *AOR* adjusted odds ratio, *CI* confidence interval

## Discussion

To the best of our knowledge, this is the first study to report on the prevalence and predictors of intestinal microsporidiosis among HIV/AIDS patients in Yemen. It revealed the prevalence of intestinal microsporidiosis among 14.2% of HIV/AIDS patients receiving treatment at the ART clinic of the NACP in Sana'a city. The prevalence of intestinal microsporidiosis in the present study is comparable to the pooled prevalence of 13.0% in Eastern Europe and Central Asia, 14.4% in Western and Central Europe and North America and 15.4% in sub-Saharan Africa [25]. Compared to the present study, prevalence rates of 11.3–16.6% among HIV/AIDS patients were reported from Mali, Venezuela, Addis Ababa of Ethiopia, Benin city of Nigeria, Chandigarh city of India and Burkina Faso (using microscopy), from Kerman city of Iran, Tunisia and Guangxi region of China (using PCR), Russia (using serological testing) and Argentina using TEM [16, 17, 30–38]. However, much lower prevalence rates of 0.8–8.5% were reported among HIV/AIDS patients from the United States of America, France, Kinshasa city of the Democratic Republic of the Congo (using PCR), Shiraz city of Iran, Bamenda city of Cameroon, Lucknow city of India (using PCR), Malaysia, the Abeokuta and Niger states of Nigeria and Hunan province of China [39–48]. On the other hand, higher prevalence rates of 21.3–85.2% were reported among HIV/AIDS patients from Uganda, Thailand, Lagos city of Nigeria, Buea and Limbe cities of Cameroon, Malawi (using PCR) and Mexico [49–54]. Variations in the prevalence of intestinal microsporidiosis could be attributed to differences in sociodemographic, environmental and behavioral risk factors. Besides, the use of different microscopical, immunological and molecular techniques plays a role in such variations. Therefore, the prevalence of intestinal microsporidiosis among HIV/AIDS patients in the present study should be compared and interpreted cautiously against the prevalence rates reported elsewhere.

The absence of a significant association between intestinal microsporidiosis and gender in the present study (18.0% vs. 13.1% for females and males, respectively) is in agreement with the finding reported among HIV-positive patients from Chandigarh city of India [17] and HIV/AIDS patients from Guangxi region of China [36]. In contrast, a significant association between intestinal microsporidiosis and gender of HIV-infected patients was reported from the Democratic Republic of the Congo, Benin city of Nigeria and Lucknow city of India [34, 41, 44]. On the other hand, the absence of a significant association between intestinal microsporidiosis and age in the present study is in agreement with the findings reported among HIV-infected patients from India, China and France [17, 36, 55]. In the present study, residence and household size were not significant predictors of intestinal microsporidiosis among HIV/AIDS patients. In contrast, a significant association was found between living in slum or rural areas and intestinal microsporidiosis among HIV-infected patients from Zimbabwe and New Delhi city of India [56, 57].

The absence of a significant association between intestinal microsporidiosis and the literacy status of HIV/AIDS patients in the present study is consistent with that reported among Chinese HIV/AIDS patients [36] and Nigerian HIV-infected patients [47]. Nevertheless, the absence of a significant association between intestinal microsporidiosis and the employment status of HIV/AIDS patients in the present study is inconsistent with that reported among Chinese HIV/AIDS patients, where the prevalence of intestinal microsporidiosis was significantly higher among farmers [36].

Having < 200 CD4 cells/µl was a significant independent predictor of intestinal microsporidiosis among HIV/AIDS patients in the present study, increasing the likelihood of infection by threefold. Therefore, physicians should consider microsporidiosis in HIV/AIDS patients with such low CD4 cell counts, particularly diarrheic patients. This finding agrees with that found among HIV/AIDS patients from the Democratic Republic of the Congo, Cameroon, Lucknow city of India, and the Abeokuta and Niger states of Nigeria [41, 44, 46, 47, 52]. In contrast, intestinal microsporidiosis was not significantly associated with CD4 cell counts among HIV/AIDS patients from the Guangxi region of China [36]. Severe immunosuppression with below 200 CD4 cells/µl increases the viral load that affects the immune system, posing patients to the risk of infection with microsporidia and other opportunistic pathogens [11]. The protective role of T-cells against microsporidia has been well documented in animal models, where mice infected with microsporidia died of the disease but survived with T-cell transfers from sensitized donor mice [58].

Diarrhea was the only gastrointestinal feature significantly associated with intestinal microsporidiosis among HIV/AIDS patients in the present study, where patients with diarrhea were 3.4-fold more likely to have intestinal microsporidiosis. Similarly, intestinal microsporidiosis was significantly associated with diarrhea among HIV-infected patients from Lucknow city of India [44]. Intestinal microsporidiosis has therefore to be considered when investigating Yemeni HIV/AIDS patients for the causes of diarrhea, considering that routine stool examination does not include the detection of microsporidia. In contrast, intestinal microsporidiosis was not significantly associated with diarrhea among HIV-infected patients from Venezuela and the Abeokuta state of Nigeria [31, 46]. In another context, intestinal microsporidiosis could not be detected using PCR among HIV-infected patients with unexplained diarrhea in Denmark [59]. The absence of a significant association between intestinal microsporidiosis and nausea, vomiting or abdominal pain among HIV/AIDS patients in the present study is in line with that observed among HIV-infected patients from Thailand [50].

The source of drinking water was not significantly associated with the acquisition of intestinal microsporidiosis in the present study, showing that HIV/AIDS patients might be more exposed to spores from other sources such as contaminated soil. However, there is a need to assess the occurrence of microsporidian spores in different water sources. Likewise, the absence of such an association was found among HIV-infected patients from France and New Delhi city of India [55, 57]. In contrast, drinking unpiped or unfiltered water was found to be significantly associated with intestinal microsporidiosis among HIV-infected patients from Lucknow city of India and Zimbabwe [44, 56].

The present finding that bathing and/or swimming outdoors was not a significant predictor of intestinal microsporidiosis HIV/AIDS patients is inconsistent with the significant association between pool swimming and intestinal microsporidiosis reported among HIV-infected patients from France [55]. Nevertheless, the present study revealed that the likelihood of infection increased by three fold and four fold, respectively, among patients not washing hands after contact with soil and those eating without washing hands. It is noteworthy that microsporidia are ubiquitous, and their spores can survive and remain infective for up to 6 months in dry conditions [60]. Soil contaminated with spores can be a major source of infection transmission through unwashed hands after contact with soil and before eating. Therefore, hand hygiene with soap and water after contact with soil and before meals is an important practice to be delivered to HIV/AIDS patients as part of educational awareness-raising messages to reduce their exposure to many foodborne infections, including intestinal microsporidiosis.

The significant association between intestinal microsporidiosis and consuming unwashed raw produce among HIV/AIDS patients in Sana’a city is consistent with that reported among Peruvian HIV/AIDS patients consuming watermelon in Lima city [61]. Microsporidia are potentially transmitted via food-borne ingestion [62], and this could partially explain why those consuming unwashed raw produce in the present study were about twice and a half more likely to have intestinal microsporidiosis compared to their counterparts. In this context, microsporidian spores have been detected on fresh fruit and vegetables, and vegetables contaminated with spores have been associated with foodborne outbreaks elsewhere [63, 64]. HIV/AIDS patients should therefore be educated about the importance of proper washing of raw produce, such as fruit and vegetables, before eating.

Microsporidia are not host-specific, infecting a wide range of invertebrate and vertebrate animals. Nevertheless, the importance of zoonotic transmission is still not fully studied, particularly in developing countries. In the present study, the presence of domestic animals was not significantly associated with intestinal microsporidiosis among HIV/AIDS patients. Consistent findings were reported among HIV/AIDS patients from France and HIV-infected patients from Lucknow city of India and Nigeria [44, 47, 55]. In contrast, the presence of domestic animals was significantly associated with intestinal microsporidiosis among HIV/AIDS patients from Zimbabwe, Lima city of Peru, New Delhi city of India and the Democratic Republic of the Congo [56, 57, 61, 65]. However, the presence of domestic animals does not mean direct exposure or contact with them, and frequent occupational contact with animals can increase the risk of infection.

The absence of indoor latrines in the present study was a significant independent predictor of intestinal microsporidiosis, where the likelihood of infection was approximately sixfold higher among those having no latrines. This finding is consistent with that observed among HIV/AIDS patients from Peru, where the lack of flush toilets in houses was significantly associated with *E. bieneusi* infection [61]. Likewise, the absence of indoor toilets and using public toilets were significantly associated with intestinal microsporidiosis among HIV-infected patients from New Delhi city of India [57]. In another context, using public and individual pit toilets was significantly associated with intestinal microsporidiosis among HIV/AIDS patients from the Democratic Republic of the Congo [65]. In contrast, the absence of latrines was not significantly associated with intestinal microsporidiosis among Nigerian HIV-infected patients [47]. Because most infections occur by human-to-human transmission through the fecal–oral route [62], the transmission of spores while using public toilets could occur through touching door handles, water taps, or using the containers that are kept in public toilets for cleaning purposes. It is noteworthy that Yemenis usually use water cups or containers instead of toilet paper for such purposes, exposing them to the risk of infection. Although indiscriminate defecation outdoors can contribute to indirect risk of infection, it was not significantly associated with intestinal microsporidiosis in the present study. In contrast, defecation in open fields was significantly associated with intestinal microsporidiosis among HIV-infected patients from New Delhi city of India [57]. Defecation in open fields could lead to contact with soil or exposure to recreational waters contaminated with spores from urine and stool of infected animals and humans [66].

The present study is limited by the use of light microscopy of stained smears for the detection of microsporidia, making their identification to the species level impossible. However, this study aimed to detect microsporidia rather than their speciation, and future molecular studies are required for the speciation and genotyping of microsporidia. This pioneer study uncovered the burden of intestinal microsporidiosis among HIV/AIDS patients in Sana’a. Another issue is that the prevalence of intestinal microsporidiosis could be higher than that found in the present study because of the lower specificity and sensitivity of light microscopy compared to molecular techniques [18]. Therefore, molecular studies are recommended to determine the prevalence of intestinal microsporidiosis among HIV/AIDS patients after controlling for the possible disadvantages of such techniques, such as the difficult extraction of spore nucleic acids and the presence of PCR inhibitors in stool samples. To reach as an accurate estimate of the prevalence of intestinal microsporidiosis as possible, the GCK staining technique was used for detecting microsporidian spores because of its high specificity and sensitivity levels compared to the chromotrope 2R-based WMT [24]. This technique is an improved modification of the quick-hot Gram-chromotrope technique [23], which was developed for rapid and good differentiation of microsporidian spores from the background fecal materials. From a diagnostic perspective, assessment of the GCK staining technique for the diagnosis of intestinal microsporidiosis needs to be evaluated compared to PCR among HIV/AIDS and other immunocompromized patients in resource-limited countries, including Yemen, where the cost and applicability of PCR are still impractical.

## Conclusions

The prevalence of intestinal microsporidiosis among HIV/AIDS patients receiving ART in Sana’a is high and comparable to that reported from several other countries, being prevalent among approximately 14.0% of patients and significantly associated with diarrhea. Therefore, special requests for its investigation in diarrheic HIV/AIDS patients by clinicians are critical, considering the impracticality of routine stool examination in its diagnosis. Demographics, source of drinking water, bathing and/or swimming outdoors, contact with soil, presence of domestic animals in the house and indiscriminate defecation could not be of value in predicting the infection among HIV/AIDS patients. However, intestinal microsporidiosis could be predicted among patients who have < 200 CD4 cells/µl, have poor hand hygiene after contacting soil and before eating, usually eat unwashed raw produce, or do not possess indoor latrines. Large-scale studies on the epidemiology and predictors of intestinal microsporidiosis among HIV/AIDS patients across the country are warranted, preferably with the adoption of molecular techniques.

## Data Availability

Data are available within the manuscript and can be provided by the corresponding author.

## Abbreviations

AIDS: Acquired immunodeficiency syndrome
AOR: Adjusted odds ratio
CD4: Cluster of differentiation 4
CI: Confidence interval
EDTA: Ethylenediaminetetraacetic acid
ELISA: Enzyme-linked immunosorbent assay
GCK: Gram-chromotrope Kinyoun
HIV: Human immunodeficiency virus
IQR: Interquartile range
NCAP: National AIDS Control Program
OR: Odds ratio
PCR: Polymerase chain reaction
SD: Standard deviation
SPSS: Statistical Package for the Social Sciences
TEM: Transmission electron microscopy
WMT: Weber’s modified trichrome
